# Gain-of-function *STAT1* mutation and visceral leishmaniasis

**DOI:** 10.31744/einstein_journal/2022RC0048

**Published:** 2022-09-05

**Authors:** Paula Teixeira Lyra, Ana Carla Augusto Moura Falcão, Rafael Amora Cruz, Antonio Victor Campos Coelho, Edvaldo da Silva Souza, Luiz Claudio Arraes de Alencar, João Bosco Oliveira

**Affiliations:** 1 Instituto de Medicina Integral Professor Fernando Figueira Recife PE Brazil Instituto de Medicina Integral Professor Fernando Figueira, Recife, PE, Brazil.; 2 Hospital Israelita Albert Einstein São Paulo SP Brazil Hospital Israelita Albert Einstein, São Paulo, SP, Brazil.

**Keywords:** STAT1 transcription factor, Germ-line mutation, Sequence analysis, DNA, Leishmaniasis, visceral, Lymphohistiocytosis, hemophagocytic

## Abstract

Gain-of-function mutations in the *STAT1* gene have been initially associated with chronic mucocutaneous candidiasis. However, further research has shown that *STAT1* GOF variants may increase susceptibility to infection by other intracellular pathogens. This report describes the first case of disseminated leishmaniasis associated with a *STAT1* GOF mutation in a pediatric patient who did not have chronic mucocutaneous candidiasis. The patient was a four-year-old boy presenting with fever, severe asthenia, hepatosplenomegaly, pancytopenia, and liver failure. Bone marrow aspirate revealed hemophagocytosis and *Leishmania* parasites. Treatment consisted primarily of liposomal amphotericin B, *as per* the Hemophagocytic Lymphohistiocytosis 2004 protocol. After eight weeks of treatment, the patient did not improve and was submitted to diagnostic splenectomy. Activated macrophages and nodular spleen necrosis secondary to the visceral leishmaniasis were detected. Unfortunately, the patient died in the second week after splenectomy due to overwhelming systemic infection. DNA sequencing revealed a pathogenic (p. R274Q) GOF mutation in *STAT1.*

## INTRODUCTION

Signal transducers and activators of transcription (STATs) are part of a family of DNA-binding proteins. These latent transcription factors are activated in the cytoplasm by the tyrosine kinase Janus kinase (JAK) to generate the mechanism of signal transduction known as the JAK-STAT pathway. Janus kinase 1 and tyrosine kinase 2 (TYK2) play a key role in the immune response and are selectively associated with the cytoplasmic domains of cytokine receptors. Binding of interleukins (IL) and interferons (IFN) to their respective receptors induces JAK activation and phosphorylate the cytoplasmic portion of the receptor, allowing selective binding of STAT proteins and their activation via phosphorylation of specific tyrosine residues. Signal transducers and activators of transcription proteins then dimerize and translocate to the nucleus, regulating gene expression.^(1-3)^

The JAK-STAT pathway was discovered during an investigation of the control of gene expression by interferons. It is now widely accepted that STATs bind to several sites in the genome, regulating thousands of genes.^(4)^ Gain-of-function (GOF) mutations were first incriminated as the cause of chronic mucocutaneous candidiasis (CMC) in 2011, with increased incidence described in some patients with autoimmune diseases, squamous cell carcinoma and intracranial aneurysm.^(5,6)^ Although more evidence is needed to support the molecular mechanisms underlying STAT1 hyperactivation in patients with *STAT1* GOF mutations,^(7)^ the lower proportion of Th17 cells often found in the peripheral blood of these patients may explain CMC development, at least in part.^(5)^

The spectrum of infections caused by the *STAT1* GOF mutations is growing and includes other fungal infections, such as coccidioidomycosis and histoplasmosis. As in loss of function (LOF) *STAT1* mutation carriers, it has been suggested that prolonged STAT1 phosphorylation may impair IFN-γ restimulation, leading to an apparent tachyphylaxis.^(8)^ Bacterial and viral infections like herpes simplex and varicella-zoster have been described in patients with immune dysregulation, polyendocrinopathy, enteropathy, X-linked (IPEX) syndrome caused by a *STAT1* GOF mutation, with or without CMC.^(9)^ In another report, this was followed by infection by *Cryptococcus* and Epstein-Barr virus.^(10)^ The expanding spectrum of clinical presentations currently comprises descriptions of recurrent infections and early autoimmunity,^(11)^ acute bronchiectasis,^(12)^ severe combined immunodeficiency (SCID),^(13-15)^ common variable immunodeficiency,^(16)^ intracranial aneurysm^(17)^ and multifocal leukoencephalopathy.^(18)^

Trichophytosis and bacterial respiratory tract infections (14% and around 40% of patients respectively) have also been reported by Depner et al.^(19)^ In a different study, Dotta et al.^(20)^ found *STAT1* GOF mutations in nine patients with CMC. Most of those patients had concurrent bacterial respiratory infections. Some presented with viral infections. A 28-year-old patient had *Cryptococcus neoformans* infection and disseminated visceral leishmaniasis, the only case described to date. In a multicenter study with 274 carriers of *STAT1* GOF variants from 40 countries, Toubiana et al.^(21)^ reported CMC in 98% of patients, bacterial infections (particularly *Staphylococcus aureus)* in 74%, viral infections (particularly by *Hesrpesviridae*) in 38%, and mycobacterial disease in 6%. Parasitic infection was also detected in two patients (giardiasis and visceral leishmaniasis, respectively), the latter extracted from Dotta et al.^(20)^ case series.

Although IFN-γ production by activated Natural Killer (NK) cells is induced by IL-12, IL-15 also plays a part in cases of infection by intracellular pathogens. Decreased proliferative response of NK cells associated with lower production of IFN-γ in response to IL-15 has been described in patients with *STAT1* GOF mutations. In cases of intracellular parasitic infections with insufficient IL-12 production to drive IFN-γ secretion by NK cells, IL-15 may be required and may explain *Cryptococcus, Leishmania* or mycobacteria infection in patients with CMC.^(22)^

This report describes and discusses the first case of visceral leishmaniasis in a patient with a *STAT1* GOF mutation who did not have CMC or any of the other previously described clinical manifestations and progressed to hemophagocytic lymphohistiocytosis and death.

## METHODS

Gene mutations were investigated using next-generation sequencing (NGS). Briefly, the sequencing library was prepared as follows: the genomic DNA was extracted from peripheral blood, quantified, and enzymatically fragmented. Sequencing adaptors were added to fragments using the SureSelectQXT Reagent kit (Agilent, Santa Clara, California, USA). DNA adaptor-tagged fragments were then amplified by multiplex polymerase chain reaction (PCR). Specific probes of the SureSelect Inherited Disease Panel (Agilent, Santa Clara, California, USA) were later hybridized with fragments from the prepared library and recovered by capture with streptavidin beads. The library was quantified using a fluorometer and pools normalized. Sequencing was performed using the Illumina NextSeq platform (Illumina, San Diego, California, USA). Raw sequencing data were processed using in-house-developed bioinformatics pipelines to generate patient variant calls.

This study adhered to the principles for research with human beings outlined in the Declaration of Helsinki and was approved by the Research Ethics Committee of *Instituto de Medicina Integral Professor Fernando Figueira* (IMIP) (# 3.044.310, CAAE: 11841312.0.0000.5201.

## RESULTS

This report describes a four-year-old Brazilian male patient born to non-consanguineous parents, so far healthy and with no relevant family health history. The patient had contact with a dog at home and was admitted with a history of fever in the last two weeks, followed by asthenia, fatigue, weight loss and myalgia. His clinical condition had deteriorated in the last few days prior to admission, with paleness, icterus, acholic stools, choluria and sleepiness. Physical examination findings were as follows: body weight of 17kg, height of 1.05m, compromised general status, pale (2/4+) and icteric (1/4+) skin and mucous membranes, sleepiness, and fever. Small, soft, painless, and mobile cervical lymph nodes were detected. Cardiac and respiratory auscultation were unremarkable. The abdomen was globose, depressible and had a tympanic sound to percussion. The liver and spleen could be palpated 10cm and 6cm below the right and left costal margins, respectively. Laboratory workup revealed pancytopenia, elevated ferritin and triglyceride levels, low fibrinogen levels, elevated liver and canalicular enzyme, bilirubin and DHL levels, and low albumin levels (Table 1). Hypocellular bone marrow, scattered *Leishmania* parasites and hemophagocytosis in numerous histiocytes were seen on the myelogram. Criteria for visceral leishmaniasis and secondary hemophagocytic lymphohistiocytosis (HLH) diagnosis were met and treatment with liposomal amphotericin B started.

**Table 1:** Laboratory workup

Test	P1	Reference range
Hemoglobin (g/dL)	8.9	
Hematocrit (%)	26	
Leukocytes (cells/mm^3^)	2100	
Neutrophils (%)	19	
Typical lymphocytes (%)	60	
Monocytes (%)	18.6	
Eosinophils (%)	0.1	
Basophils (%)	2.7	
Platelets (10^3^/mL)	86	
Ferritin (ng/mL)	>1650	30-200
Triglycerides (mg/dL)	627	
Fibrinogen (mg/dL)	170	180-350
Aspartate aminotransferase (U/L)	882	0-35
Alanine aminotransferase (U/L)	308	0-35
Total bilirubin (mg/dL)	5.3	0.3-1.2
Indirect bilirubin (mg/dL)	4.9	
Direct bilirubin (mg/dL)	0.4	0-0.3
Lactate dehydrogenase (U/L)	6170	60-160
Albumin (m/dL)	1.4	3.5-5.4
Alkaline phosphatase (U/L)	968	36-150
Amylase (U/L)	223	25-125
Soluble CD25	Not measured	
NK cell activity	Not assessed	
IgA (mg/dL)	190	
IgG (mg/dL)	1600	
IgM (mg/dL)	83	
IgE (UI/L)	148	
Rubella IgG	Reactive	
HIV Serology	Non-reactive	
Immunophenotyping of lymphocytes	Not performed	
Rectal swab specimen culture	Multi-resistant *Klebsiella pneumoniae,* sensitive to polymyxin B	
Blood culture	Negative	

NK: natural killer; IgA: immunoglobulin A; IgG: immunoglobulin G; IgM: immunoglobulin M: IgE: immunoglobulin E.

The patient progressed to respiratory failure and shock requiring assisted mechanical ventilation and vasoactive drugs. Blood and gastric lavage cultures were negative for mycobacteria. Tuberculin hypersensitivity skin test results were also negative (0 mm). There was no significant clinical or laboratory improvement after treatment with amphotericin B. Treatment with dexamethasone, cyclosporin and intravenous human immunoglobulin was then introduced *as per* the HLH-2004 protocol,^(23)^ except for etoposide.

The patient developed acute abdomen (partial intestinal obstruction and unspecified peritonitis) and was submitted to exploratory laparotomy, which revealed significant amounts of bilious fluid, small bowel bridles, fibrin deposition in the paracolic gutter and hyperemic small bowel loops and colon. *Staphylococcus epidermidis* and *S. haemolyticus* infection occurred after surgery. Clinical and laboratory signs of active HLH persisted, with acute neutropenia and significant splenomegaly eight weeks after introduction of the HLH treatment protocol. Abdominal ultrasound revealed homogenous hepatomegaly and heterogeneous splenomegaly with numerous focal, oval hypoechoic splenic lesions with no flow on Doppler. Multiple hypodense splenic lesions were found on computed tomography (CT). Chest CT was carried out due to persistent dyspnea and hypoxemia requiring oxygen therapy, but failed to reveal signs of pulmonary disease. The patient developed acute renal injury and was kept on long-term parenteral nutrition, since nasogastric tube feeding attempts were unsuccessful. The hepatic injury worsened, as shown by elevated transaminases and canalicular enzymes, abdominal distention, and painful hepatosplenomegaly. Abdominal radiography revealed lack of gas.

Diagnostic splenectomy was suggested after case discussion with the pediatric surgery team. Prior to surgery, the patient was readmitted to the intensive care unit due to idiopathic infection and respiratory failure, with persistent fever and condensation in the left hemithorax. Treatment consisted of vancomycin (28 days), linezolid, meropenem (14 days), amphotericin B (28 days) and amikacin, which was switched to ciprofloxacin due to acute pancreatitis.

Splenectomy findings (five months after hospital admission) included an enlarged heterogeneous spleen with multiple nodulations, numerous adhesions to the posterior abdominal wall, diaphragm and tail of the pancreas, and lesser amounts of ascitic fluid. Four additional surgical interventions were needed for the following reasons: considerable build-up of bloody fluid in the abdominal cavity, with obstruction of the jejunum, “dripping candle wax”-like implants in the omentum and abdominal wall and hardening of the tail of the pancreas (3^rd^ day post-splenectomy), necrotizing pancreatitis (6^th^ day post-splenectomy), surgical wound abscess (9^th^ day post-splenectomy) and peritonitis (11^th^ day post-splenectomy). The patient died on the 12^th^ day post-splenectomy, presumably due to sepsis while receiving polymyxin B.

Gene sequencing revealed a c.821G>A (p.R274Q) heterozygous pathogenic GOF mutation in exon 10 of the coiled-coil (CC) domain of *STAT1*.

## DISCUSSION

This report describes a patient with a heterozygous GOF mutation in the CC domain of *STAT1*. The initial clinical presentation consisted of visceral leishmaniasis without concurrent CMC. This mutation had already been described in patients with CMC^(19,24)^ and other fungal, viral, or bacterial infections^(25)^ and in patients with progressive multifocal leukoencephalopathy.^(18)^ Surprisingly, asymptomatic carriers of the same mutation have been identified in other studies.

In the most comprehensive international study to date, CMC was reported in 98% of patients with GOF mutations in *STAT1*. However, that study also emphasized that these mutations underlie a broad clinical phenotype, with potential overlap of primary manifestations. *STAT1* GOF mutations are found in approximately half of patients with CMC.^(21)^ Therefore, such mutations are not expected in patients who do not have CMC, as the one reported in this study.

The STAT1 protein consists of numerous domains: N-terminal (NT), coiled-coil (CC), DNA-binding (DNA-B), linker (L), Src homology 2 (SH2), tail segment (TS), and transactivation (TA).^(26)^ In patients with CMC, *STAT1* GOF mutations have been more often identified in the CC and DNA-B domains.^(27)^ However, these mutations have also recently been detected in the TA, NT and SH2 domains.^(21,28)^

Visceral leishmaniasis is the more acute systemic form of leishmaniasis and is usually fatal if left untreated. In Brazil, the disease is endemic and caused by *Leishmania infantum*. Fever and splenomegaly are the typical clinical signs, although pancytopenia, hepatomegaly, weight loss and hypergammaglobulinemia are also common,^(29)^ as in the case described. Hemophagocytic lymphohistiocytosis, a secondary hyperinflammation syndrome resulting from the secretion of elevated levels of pro-inflammatory cytokines, is a potential complication of visceral leishmaniasis.^(30)^ The diagnosis of HLH is based on at least five out of eight criteria listed in the HLH-2004 protocol (Table 2). The patient described in this report met HLH diagnostic criteria due to inadequate response to visceral leishmaniasis treatment with liposomal amphotericin B, as in prior cases of HLH secondary to visceral leishmaniasis. In the case reported, therapy was established *as per* the HLH-2004 protocol. Unfavorable clinical progression led to diagnostic splenectomy, with findings consistent with visceral leishmaniasis and hemophagocytic activity. Unfortunately, gene sequencing was only available after the patient’s death. His immunization schedule is shown in table 3.

**Table 2:** Hemophagocytic lymphohistiocytosis (HLH-2004) diagnostic criteria

Criteria	P1
Clinical criteria	
Fever	Yes
Splenomegaly	Yes
Laboratory criteria	
Cytopenia (affecting two out of three cell populations)	Yes
Hypertriglyceridemia or hypofibrinogenemia	Yes
Hyperferritinemia >500	Yes
Decreased or absent NK cell activity	Not assessed
Elevated soluble CD25 level (>2400 U/mL)	Not measured
Histopathological criteria	
Hemophagocytosis in bone marrow, spleen, or lymph nodes	Yes
No evidence of malignancy	Yes

NK: natural killer.

**Table 3:** Vaccines administered to the patient

Vaccine	Primary doses + booster doses	Age (in months unless otherwise stated)
Bacillus Calmette-Guérin (BCG)	1	1 day
Hepatitis B	4	1 day, 1 and 6
Diphtheria, pertussis, and tetanus (DPT)	3 + 2	2, 4, 6, 15 and 48
*Haemophilus influenzae* type B (Hib)	3	2, 4 and 6
Oral polio	3 + 1	2, 4, 6 and 15
Oral human rotavirus	2	2 and 4
10-valent pneumococcal conjugate (PCV10)	3 + 1	2, 4, 6 and 12
Meningococcal group C conjugate	2 + 1	3, 5 and 15
Measles, mumps, and rubella (MMR)	2	12 and 48

Reduced IFN-γ production by activated NK cells in response to IL-15 may explain parasitic infections in patients with GOF mutations in *STAT1*.^(22)^ In the case described, functional analysis of NK cells could not be performed due to delayed postmortem molecular diagnosis. Mutations in the DNA-B and CC domains of *STAT1* in patients with CMC and immunodeficiency phenotype combined with HLH have only been described in the context of viral infections. Mechanisms underlying HLH in patients with *STAT1* GOF mutations remain to be elucidated. However, defective NK cell cytotoxicity may increase the risk of HLH, particularly when associated with viral infections.^(22,25)^

## CONCLUSION

This report describes the first case of hemophagocytic lymphohistiocytosis secondary to visceral leishmaniasis in a patient with a heterozygous gain-of-function mutation in *STAT1* who did not have chronic mucocutaneous candidiasis.
